# Switching to Faricimab in Therapy-Resistant Macular Edema Due to Retinal Vein Occlusion: Initial Real-World Efficacy Outcomes

**DOI:** 10.3390/jcm14072454

**Published:** 2025-04-03

**Authors:** Michael Hafner, Tina R. Herold, Alexander Kufner, Ben Asani, Andreas Anschütz, Franziska Eckardt, Siegfried G. Priglinger, Johannes Schiefelbein

**Affiliations:** Department of Ophthalmology, LMU University Hospital, LMU Munich, 80336 Munich, Germany

**Keywords:** Faricimab, retinal vein occlusion, macular edema, VEGF, angiopoietin-2, intravitreal therapy, optical coherence tomography

## Abstract

**Background/Objectives**: Macular edema (ME), due to retinal vein occlusion (RVO), is a major cause of vision impairment. Many patients experience suboptimal responses to anti-vascular endothelial growth factor (anti-VEGF) monotherapy, necessitating alternative treatment approaches. Faricimab, a bispecific antibody targeting VEGF-A and angiopoietin-2 (Ang-2), introduces a novel dual-mechanism therapy. This study evaluates the short-term real-world efficacy of switching to Faricimab in patients with treatment-resistant ME secondary to RVO. **Methods**: This retrospective study included patients from LMU University Hospital who were switched to Faricimab due to an inadequate response or adverse events related to prior intravitreal therapy (Ranibizumab, Aflibercept, or Ozurdex^TM^). All patients completed a structured loading phase of four monthly injections. Key outcome measures included changes in best-corrected visual acuity (BCVA, logMAR), central subfield thickness (CST, µm), and intraretinal fluid (IRF) presence on optical coherence tomography (OCT). Changes were assessed from baseline (mo0) to three months (mo3). **Results:** The study included 19 eyes from 19 patients (mean age 63.0 ± 14.2 years). BCVA improved from 0.20 logMAR at baseline to 0.00 logMAR at mo3 (*p* < 0.01). CST decreased from 325 µm to 280 µm (*p* < 0.01). The proportion of eyes with IRF reduced from 100% to 32% (*p* < 0.01). Significant reductions in retinal volume within the 1 mm and 6 mm (both *p* < 0.01) circles of the ETDRS grid were observed. **Conclusions**: Switching to Faricimab in patients resulted in significant short-term improvements in BCVA, CST, and IRF resolution. Given the small sample size and retrospective design, these findings should be interpreted as exploratory and hypothesis-generating. Further studies are needed to evaluate long-term efficacy and optimal treatment regimens.

## 1. Introduction

As a common and sight-threatening complication of retinal vein occlusion (RVO), macular edema (ME) remains a leading cause of vision impairment worldwide [1,2]. The development of ME in RVO results from disrupted vascular integrity and increased permeability of the retinal blood vessels, leading to vascular leakage and fluid accumulation in the macula and, as a result, a reduction in visual acuity [3]. Given the progressive nature of this condition, refining treatment strategies is essential to prevent irreversible vision loss and to ensure long-term visual function.

The current cornerstone of ME management in RVO involves intravitreal anti-vascular endothelial growth factor (anti-VEGF) therapies, which effectively target VEGF-induced vascular leakage and promote stabilization of retinal vasculature. In addition, dexamethasone implants (Ozurdex^TM^, Allergan^®^) are frequently used due to their broad anti-inflammatory effects, which often lead to good therapeutic responses [4]. Their depot effect also reduces the number of required injections, offering a practical advantage in long-term management [5]. However, dexamethasone implants are not suitable for all patients, as their use is limited by potential adverse effects such as steroid-induced intraocular pressure (IOP) elevation, cataract progression in phakic patients, and, in rare cases, anterior chamber dislocation [5,6]. Due to these limitations of dexamethasone implants, anti-VEGF therapies often remain a necessary therapeutic strategy in clinical practice. While clinical trials consistently demonstrate the efficacy of these therapeutic agents, translating the results into real-world clinical practice has proven challenging. Inconsistent treatment adherence and resistance to therapy often compromise the ability to achieve similar outcomes, leaving a subset of patients with persistent or recurrent ME [7,8].

Emerging research underscores the complex and multifactorial nature of ME pathophysiology in RVO. Beyond VEGF-driven vascular permeability, elevated levels of angiopoietin-2 (Ang-2) have been implicated in the progression of ME [9]. Ang-2 competes with angiopoietin-1 for binding to the Tie2 receptor, a signaling pathway critical for maintaining vascular integrity. By antagonizing the stabilizing effects of angiopoietin-1, Ang-2 exacerbates vascular instability, promoting further leakage, inflammation, and disease progression [10]. These findings suggest that VEGF inhibition alone may not adequately address all aspects of ME pathophysiology, necessitating therapeutic strategies that also target other molecular pathways. Preclinical studies indicate that dual inhibition of VEGF and Ang-2 has the potential to stabilize blood vessels, reduce inflammation, and control pathological neovascularization, offering a more comprehensive approach to managing ME [11].

Faricimab (Vabysmo^®^, Roche/Genentech), a bispecific monoclonal antibody, simultaneously inhibits VEGF-A and Ang-2, introducing a novel dual-targeting mechanism for the treatment of ME [12]. Its efficacy and safety have been extensively validated in large-scale clinical trials. The YOSEMITE and RHINE trials demonstrated Faricimab’s therapeutic potential in managing Diabetic Macular Edema (DME) [13], while the TENAYA and LUCERNE trials established its non-inferiority to Aflibercept in treating neovascular age-related macular degeneration (nAMD) [14]. Building on this foundation, the BALATON and COMINO studies confirmed the efficacy of Faricimab in ME caused by RVO, demonstrating visual and anatomical improvements comparable to Aflibercept [15].

Despite these encouraging clinical trial results, real-world applicability remains a critical area of investigation, particularly for patients with treatment-resistant ME. Clinical trials often operate under controlled conditions that may not fully capture the complexities of real-world patient populations, including those with variable comorbidities, advanced disease stages, or limited adherence and compliance to treatment schedules [16]. This study seeks to bridge this gap by evaluating the real-world impact of Faricimab in patients with recalcitrant ME secondary to RVO. Specifically, it focuses on short-term outcomes following a structured loading phase of four intravitreal injections administered at four-week intervals, mirroring the regimen established for nAMD treatment. Key parameters assessed include improvements in best-corrected visual acuity (BCVA), reductions in central subfield thickness (CST), and resolution of fluid on optical coherence tomography (OCT).

OCT is a non-invasive imaging technique that has become an essential tool in diagnosing and monitoring various systemic and local diseases. It allows for the precise evaluation of retinal structures, facilitating accurate assessment and monitoring of disease progression and response to treatment. OCT has been widely used to detect alterations in various retinal pathologies [17].

## 2. Materials and Methods

### 2.1. Participants

This retrospective analysis used the Smart Eye Database by the Department of Ophthalmology at LMU University Hospital Munich to identify and evaluate patients treated with Faricimab for RVO during the period from July 2024 to March 2025. The inclusion criteria were defined as follows: (i) patients were switched to Faricimab therapy due to suboptimal outcomes with prior treatments, including Ranibizumab, Aflibercept, or dexamethasone intravitreal implant. Treatment resistance was defined based on the presence of persistent or recurrent IRF despite consistent anti-VEGF or corticosteroid therapy. Patients were considered treatment-resistant if they exhibited persistent IRF despite regular monthly anti-VEGF injections (Ranibizumab, Aflibercept) over a minimum period of three months, or if they were unable to extend treatment intervals beyond six weeks without fluid recurrence by the seventh week in a treat-and-extend regimen. For patients treated with dexamethasone intravitreal implant, treatment resistance was defined as persistent IRF despite intravitreal corticosteroid therapy, or recurrence of fluid before 90 days following a corticosteroid injection where longer-lasting control was anticipated. Additionally, documented adverse effects related to corticosteroid therapy, such as steroid-induced IOP elevation, cataract progression in phakic patients, or anterior chamber dislocation, were considered indications for switching to Faricimab [5,6]; (ii) patients had completed a loading phase of four consecutive Faricimab injections within a period of 3 ± 1 months; (iii) patients had no concurrent conditions, such as intraocular infections, uveitis or other retinal or macular diseases. Eyes with other macular diseases responsible for the ME were excluded. These conditions included vitreomacular traction, diabetic ME, postoperative ME, macular telangiectasia, macular aneurysm, vitreous hemorrhage, or uncontrolled glaucoma characterized by IOP exceeding 30 mmHg, as measured using non-contact tonometry.

The study was conducted in accordance with the Declaration of Helsinki. Ethical review and approval were waived by the Institutional Review Board of the Faculty of Medicine at LMU Munich (ID: 25-0070 KB). A range of demographic and clinical data was systematically collected for each patient, including baseline information such as age and gender, the date of initial intravitreal therapy, the number and type of prior injections administered, and the date of medication switch to Faricimab.

This study explored the practical application of Faricimab in addressing persistent or recurrent fluid accumulation, focusing on its potential efficacy in patients with extensive treatment histories where conventional therapies had proven insufficient.

### 2.2. Intravitreal Treatment

A structured loading phase of four Faricimab injections was administered at intervals of at least 28 days (approximately one per month) over a period of 3 (±1) months for rapid disease stabilization, followed by individualized interval extensions based on clinical response.

### 2.3. Pre-Treatment Evaluations

Pre-treatment assessments included the measurement of BCVA, which began with obtaining autorefractor readings using a Nidek^®^ AR-1s device. Subsequently, BCVA was determined through subjective refinement of these values, using a Snellen chart positioned at a distance of 4 m. IOP was evaluated via non-contact tonometry (Nidek^®^ REF/KERATO/TONOMETER Tonoref II), and a dilated fundus examination was performed. Macular anatomy and fluid accumulation were routinely assessed with SD-OCT and near-infrared imaging using the Spectralis HRA+OCT system (Heidelberg Engineering). RVO diagnosis was initially confirmed with OCT and ultrawide-field fluorescein angiography.

Quantitative metrics, such as CST and retinal volume, were analyzed with Heidelberg Eye Explorer software (Version 1.10.12.0), with manual corrections applied as needed. Values are given with regard to the established ETDRS grid circles [18]. Baseline OCT data (mo0) and follow-up scans during the loading phase (mo1, mo2, and mo3) were systematically collected.

### 2.4. Data Management and Statistical Analysis

Collected data were managed using Microsoft Excel (Version 16.78.3 for Mac). Statistical evaluations were conducted using GraphPad Prism (Version 10.3.1 for macOS). A significance level of *p* < 0.05 was applied. The Shapiro–Wilk test revealed a non-normal distribution of data, prompting the use of non-parametric statistical methods. Changes in key biomarkers, including CST and BCVA, from baseline (mo0) to the end of the loading phase (mo3), were evaluated using the Wilcoxon matched-pairs signed-rank test. Differences in the proportion of eyes with IRF between mo0 and mo3 were analyzed using McNemar’s test via GraphPad’s online calculator.

## 3. Results

### 3.1. Baseline Demographics

This study included 19 eyes from 19 patients with treatment-resistant ME secondary to RVO. Demographic and clinical characteristics are detailed in Table 1.

The mean age of patients at the time of switching to Faricimab was 63.0 ± 14.2 years (mean ± standard deviation), with a gender distribution of twelve males (63%) and seven females (37%). Patients had received an average of 35.1 ± 27.2 intravitreal injections before switching to Faricimab, including 16.8 ± 22.7 Ranibizumab, 17.2 ± 25.9 Aflibercept, and 1.1 ± 3.3 Ozurdex^TM^ injections. The annual injection rate averaged 9.6 ± 3.0, reflecting the heavy treatment burden. At the last injection before switching to Faricimab, eight patients were on Ranibizumab, nine on Aflibercept, and two on Ozurdex^TM^.

Prior to the switch, retinal laser photocoagulation with the PASCAL (Optimedica Corp., Santa Clara, CA, USA) or Navilas 577s (OD-OS GmbH, Teltow, Germany) system was applied in 10 patients who had responded particularly poorly to previous therapies. Ultrawide-field fluorescein angiography was used to identify ischemic areas after RVO, allowing for targeted sectoral retinal laser treatment in these regions. The goal of this intervention was to potentially reduce the frequency of intravitreal injections by addressing the ongoing VEGF drive from ischemic retinal zones [19]. This underscores the complexity of the patient cohort analyzed and the ongoing need for alternative strategies in cases where anti-VEGF or corticosteroid monotherapy proves insufficient for long-term disease control.

### 3.2. Visual Acuity (BCVA)

**Figure 1:**
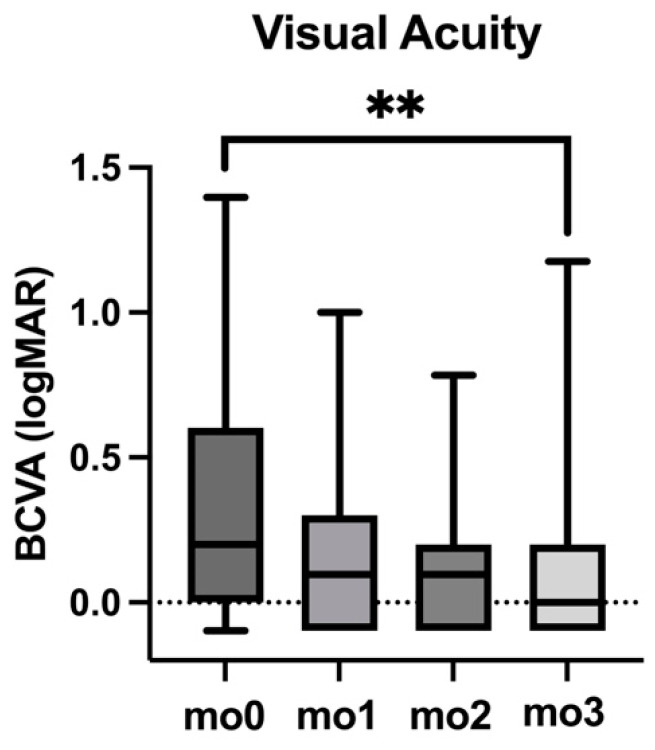
Best-corrected visual acuity (BCVA) changes during the loading phase of the Faricimab treatment between baseline (mo0) and follow-up visits (mo1, mo2, and mo3). *p*-value is indicated by asterisks (with ** as *p* < 0.01).

BCVA showed significant improvement throughout the loading phase with Faricimab. At baseline (mo0), the median BCVA was 0.20 logMAR, with an interquartile range (IQR) of 0.60. By the first follow-up (mo1), BCVA improved to 0.10 logMAR (IQR: 0.40), and was 0.10 logMAR (IQR: 0.30) at mo2. By the end of the loading phase (mo3), BCVA had further improved to 0.00 logMAR (IQR: 0.30). The improvement in BCVA from mo0 to mo3 was statistically significant (*p* < 0.01). Data are detailed in Table 2 and Figure 1.

### 3.3. Central Subfield Thickness

**Figure 2:**
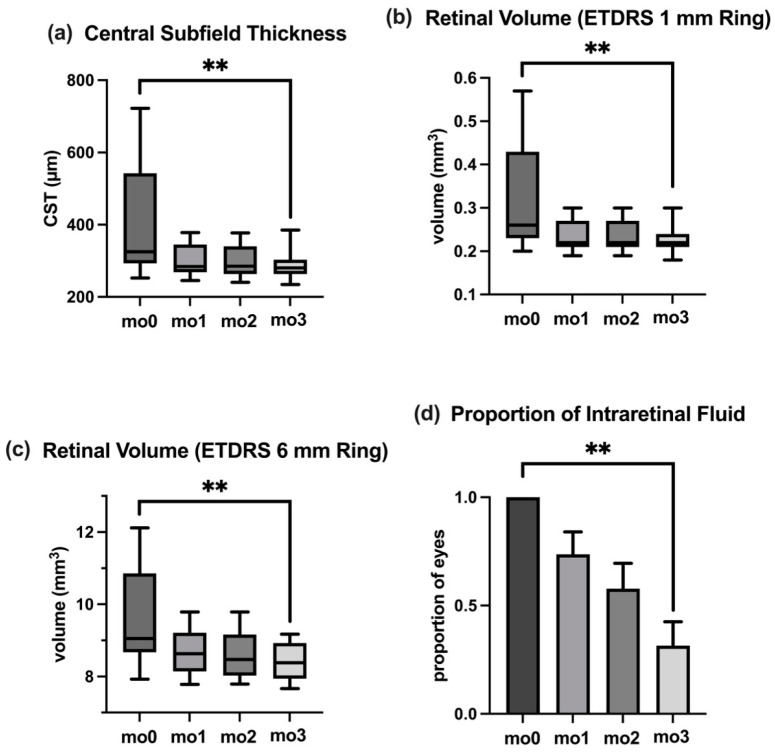
Temporal changes in OCT biomarkers during the loading phase of the Faricimab treatment between baseline (mo0) and follow-up visits (mo1, mo2, mo3). (**a**) Central subfield thickness (CST). (**b**) Retinal volume in ETDRS 1 mm ring. (**c**) Retinal volume in ETDRS 6 mm ring. (**d**) Proportion of eyes with intraretinal fluid (IRF). *p*-values are indicated by asterisks (with ** as *p* < 0.01).

CST, a critical marker for ME, showed substantial improvement over the course of the study. The median CST at baseline (mo0) was 325 (IQR: 249) µm, reflecting significant macular swelling. This decreased to 284 (IQR: 77) µm at mo1, and further reduced to 278 (IQR: 79) µm at mo2 and 280 (IQR: 40) µm at mo3. The reduction in CST from baseline to mo3 was highly significant (*p* < 0.01). Data are shown in Table 2 and Figure 2a.

### 3.4. Retinal Volume

Reductions in retinal volume further reinforced the anatomical efficacy of Faricimab. Within the 1 mm ETDRS grid circle, retinal volume decreased significantly from 0.26 (IQR: 0.20) mm^3^ at baseline to 0.22 (IQR: 0.03) mm^3^ at mo3 (*p* < 0.01). Similarly, the 6 mm ETDRS grid circle volume reduced from 9.05 (IQR: 2.19) mm^3^ at baseline to 8.38 (IQR: 0.99) mm^3^ at mo3 (*p* < 0.01). Data are presented in Table 2 and Figure 2b,c.

### 3.5. Intraocular Pressure

IOP remained stable over the course of the study, with no significant changes noted. The median IOP at baseline (mo0) was 16 (IQR: 3) mmHg. Median IOP remained unchanged at mo1, mo2, and mo3. Data are presented in Table 1.

The stable IOP values throughout the study period suggest that Faricimab does not pose a risk for pressure-related complications, affirming its safety profile in this patient cohort.

### 3.6. Intraretinal Fluid

The proportion of eyes with IRF decreased significantly during the study. At baseline (mo0), 100% of eyes exhibited IRF, with a standard error of the mean (SEM) of 0%. By mo1, this proportion reduced to 74% (SEM: 10%), and continued to decline at mo2 to 58% (SEM: 12%). By the final assessment at mo3, only 32% of eyes exhibited IRF (SEM: 11%), representing a significant reduction from baseline (*p* < 0.01). Data are detailed in Table 2 and Figure 2d.

**Figure 3:**
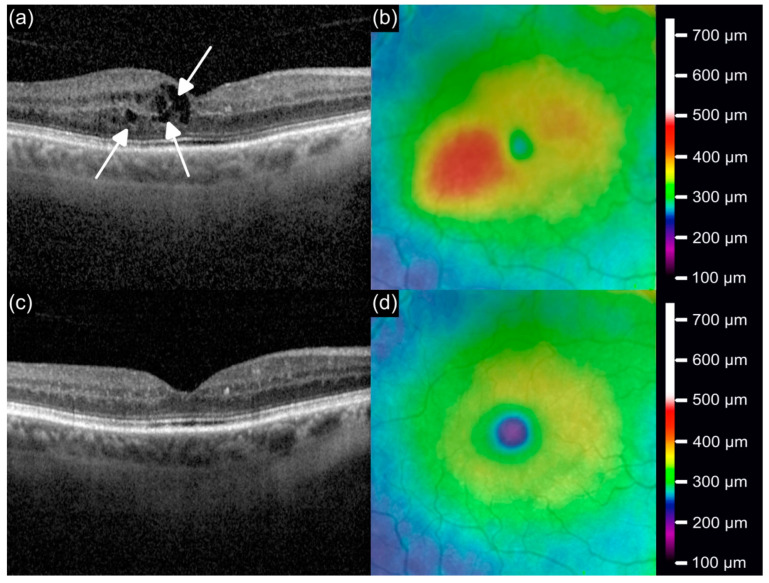
OCT and thickness map images illustrating macular anatomy at mo0 before the switch to Faricimab (**a**,**b**) and at mo3 (**c**,**d**). (**a**) Baseline OCT scan (mo0) showing substantial intraretinal fluid accumulation (indicated by arrows), with increased central subfield thickness. (**b**) Corresponding thickness map depicting elevated retinal thickness (red region). (**c**) Follow-up OCT scan (mo3) demonstrating reduction in intraretinal fluid with restoration of macular architecture after treatment with Faricimab. (**d**) Corresponding thickness map illustrating normalized retinal thickness with reduced central subfield thickness.

An example for the reduction in IRF and CST after switch to Faricimab is shown in Figure 3. At baseline (mo0; Figure 3a,b), the OCT scan reveals IRF accumulation and increased CST, reflecting active disease. By the three-month follow-up (mo3; Figure 3c,d), the OCT scan shows a substantial reduction in intraretinal fluid, restoration of macular architecture, and a marked reduction in CST, underscoring the anatomical efficacy of the Faricimab treatment.

### 3.7. Correlation Analysis Between BCVA and Anatomical Parameters

The analysis demonstrated a strong positive correlation between BCVA (logMAR) and key anatomical markers of ME, including CST and retinal volumes within the 1 mm and 6 mm ETDRS grid circles. The correlation coefficients (Spearman’s r) were 0.58 for CST, 0.57 for the 1 mm ETDRS circle volume, and 0.49 for the 6 mm ETDRS circle volume. All correlations were statistically significant (CST: *p* < 0.01, ETDRS 1 mm: *p* < 0.01, and ETDRS 6 mm: *p* = 0.02, respectively).

Regression analysis further quantified these relationships. For CST, each unit increase resulted in a BCVA change of 0.0019 logMAR/µm, with a y-intercept of −0.43 logMAR. For example, a CST reduction of 100 µm would correspond to a BCVA improvement of 0.19 logMAR, equivalent to a gain of approximately 9.5 ETDRS letters.

For the 1 mm ETDRS circle volume, a slope of 2.4180 logMAR/mm^3^ indicates a greater visual improvement associated with volume reduction compared to the 6 mm circle volume slope of 0.1616 logMAR/mm^3^. Data are presented in Table 3.

## 4. Discussion

The present study aimed to address the gap between clinical trials and real-world data by evaluating the short-term response to Faricimab in patients who were switched from prior intravitreal treatments due to inadequate treatment response after multiple intravitreal injections of different medications. Treatment resistance was defined as persistent intraretinal fluid affecting BCVA despite consistent 4-weekly dosing of anti-VEGF or the inability to extend treat-and-extend intervals beyond an interval of 6 weeks (or 90 days for Ozurdex^TM^). Moreover, adverse effects of Ozurdex^TM^ had to be taken into account, such as the risk of cataract progression in phakic patients, steroid-induced IOP elevation, or anterior chamber dislocation [5,6].

To the best of our knowledge, this study is the first to analyze recalcitrant RVO patients switched to Faricimab in a real-world setting.

Our study included 19 eyes from 19 patients with treatment-resistant ME secondary to RVO. The baseline demographics, marked by extensive prior treatment histories with various intravitreal agents, underscore the complexity of managing refractory ME and emphasize the critical need for innovative therapies like Faricimab in this challenging population.

A significant improvement in BCVA was observed during the study, indicating that the applied treatment approach was effective in enhancing the functional outcomes for patients. This marked improvement aligns with Faricimab’s dual mechanism of action, which targets both VEGF and Ang-2, addressing the multifactorial pathophysiology of RVO-associated ME [10].

Anatomical improvements were evident, with significant reductions in CST and retinal volume within both the 1 mm and 6 mm ETDRS grid circles by mo3, reflecting Faricimab’s efficacy in reducing macular swelling and restoring retinal structure.

IRF, a key indicator of disease activity, resolved significantly in a majority of eyes. At baseline, 100% of eyes exhibited IRF, which was reduced to 32% by mo3 (*p* < 0.01). The resolution of IRF is particularly critical for visual recovery, as fluid is associated with poor visual outcomes [20]. Additionally, IOP remained stable throughout the study, affirming Faricimab’s favorable safety profile for long-term use in refractory ME.

Our findings emphasize Faricimab’s ability to address the limitations of VEGF monotherapy. For example, the RELATE study demonstrated no clinically significant benefit from increasing the Ranibizumab dose to 2.0 mg compared to the standard 0.5 mg in managing ME secondary to RVO [21]. This suggests that merely increasing the dosage of anti-VEGF agents may not provide additional therapeutic advantages. However, the dual-targeting mechanism of Faricimab, which inhibits both VEGF and Ang-2, offers a more comprehensive strategy that could enhance efficacy, particularly in patients with treatment-resistant ME.

At baseline (mo0), our study demonstrated a significant correlation between BCVA (logMAR) and CST, as well as retinal volumes within the 1 mm and 6 mm ETDRS grid circles. In our cohort of treatment-resistant ME patients, a 100 µm increase in CST before the switch to Faricimab was associated with a BCVA decline of 0.19 logMAR (approximately 9.5 ETDRS letters). This slope is notably steeper than the 3.2 ETDRS letter loss per 100 µm increase reported by Michl et al. [20] in their treatment-naïve RVO cohort.

This disparity likely reflects differences in patient populations. Unlike the treatment-naïve cohort studied by Michl et al., our patients had a long history of persistent ME and prior anti-VEGF therapy, leading to chronic damage and a stronger impact of persistent or recurring higher CST values on BCVA. The long-standing nature of the edema in our cohort likely exacerbated photoreceptor damage and structural disorganization, intensifying the functional consequences of CST increases [22].

Our study demonstrated a stronger correlation between BCVA and the 1 mm ETDRS circle volume (r = 0.57) compared to the 6 mm ETDRS circle volume (r = 0.49), emphasizing the critical importance of the central macula in visual acuity. Regression analysis further supported this, showing that for the 1 mm ETDRS circle volume, a reduction of 1 mm^3^ in retinal volume correlated with a BCVA improvement of approximately 2.4180 logMAR. In contrast, the slope for the 6 mm ETDRS circle volume was 0.1616 logMAR/mm^3^, indicating a smaller visual impact for the same volumetric reduction. These findings highlight the localized impact of central ME on visual acuity, with changes in the central macula (1 mm grid) contributing more to visual function than those in the peripheral macula (represented by the 6 mm grid).

In the real-world setting, our study highlights the utility of Faricimab in patients who had previously failed multiple lines of therapy. Unlike controlled clinical trials, real-world studies often capture a more diverse patient population with varying degrees of disease severity and comorbidities [23]. This adds to the clinical relevance of our findings and provides insights into the practical application of Faricimab in complex cases.

However, our study has several limitations, including its retrospective design and relatively small sample size.

The relatively small sample size (n = 19) limits both the statistical power and the generalizability of our findings. This reflects the limited availability of real-world data, as Faricimab was only recently approved for RVO treatment in the European Union. Moreover, the retrospective nature of the study and the absence of a control group prevent any definitive causal inference, as improvements observed following the switch to Faricimab may also be influenced by confounding factors. The heterogeneous baseline characteristics of the cohort—including variability in treatment histories, disease duration, and prior interventions—further complicate the interpretation of the pooled results. Due to the limited sample size and associated lack of statistical power, stratified analyses or subgroup comparisons were not feasible. These limitations highlight the need for caution when interpreting the results. Therefore, the outcomes reported here should be regarded as exploratory and hypothesis-generating. They provide preliminary insights into the potential benefits of Faricimab in a treatment-resistant RVO population but require confirmation through larger, prospective, controlled trials with standardized baseline criteria to better assess true efficacy and treatment durability.

Additionally, the follow-up period was limited to three months, precluding long-term evaluations of efficacy and safety. While the short-term efficacy of Faricimab is promising, long-term studies are crucial to determine its durability and optimal use in clinical practice.

One key area of interest is the potential to extend treatment intervals within a treat-and-extend regimen [24]. The dual inhibition mechanism of VEGF and Ang-2 by Faricimab might allow for greater treatment durability compared to VEGF monotherapy, as has already been shown for nAMD in a real-world setting [25]. However, robust evidence is needed to evaluate whether Faricimab can consistently maintain anatomical and functional improvements over extended intervals in patients with treatment-resistant ME due to RVO. Future research should aim to investigate the extent to which Faricimab allows for longer treatment intervals compared to other intravitreal therapies.

In the pivotal BALATON and COMINO trials, six intravitreal injections of Faricimab were administered at 4-week intervals during the initial treatment phase [15]. This approach aimed to establish robust disease control before extending treatment intervals.

In contrast, our study employed a loading phase of four intravitreal injections at 4-week intervals. The decision to implement a four-injection loading phase was based on both practical and clinical considerations. First, our study focused exclusively on patients with treatment-resistant ME who had already undergone extensive anti-VEGF and/or corticosteroid therapy prior to switching to Faricimab. Unlike treatment-naïve patients included in the pivotal trials, this cohort demonstrated persistent or recurrent disease activity despite high treatment intensity. Given this context, a shorter loading phase was considered appropriate to evaluate early treatment response without further extending the already high treatment burden. Second, this approach aligns with the established loading protocol for nAMD [14], where four monthly initial injections are considered sufficient for disease control. Reducing the number of initial injections also reflects a patient-centered strategy aimed at minimizing treatment fatigue, improving adherence, and optimizing clinic resources. Despite the shorter loading phase, our results demonstrated strong anatomical and functional outcomes, prompting important questions about the necessity and optimal length of the loading phase across different indications. Future prospective studies are needed to compare loading strategies across different patient populations, determining the optimal initiation protocol for Faricimab in treatment-resistant ME due to RVO.

In conclusion, the dual inhibition of VEGF and Ang-2 by Faricimab represents a promising advancement in the treatment of resistant ME secondary to RVO. However, the short-term follow-up period of 3 months limits the ability to draw conclusions regarding the long-term durability of Faricimab treatment and the potential for interval extension. Future investigations should explore the long-term efficacy and safety of Faricimab and its role in optimizing treatment regimens for retinal vascular diseases.

## Figures and Tables

**Table 1:** Baseline demographics and treatment history of the study cohort.

Number of patients	19
Number of eyes	19
Mean age (years)	63.0 ± 14.2
Gender	
Male	12
Female	7
Mean intravitreal injections prior to switch to Faricimab	
Total (*n*)	35.1 ± 27.2
Total Ranibizumab	16.8 ± 22.7
Total Aflibercept	17.2 ± 25.9
Total dexamethasone implants (Ozurdex^TM^)	1.1 ± 3.3
Mean injections per year prior to switch to Faricimab	9.6 ± 3.0
Last injection prior to switch to Faricimab	
Ranibizumab	8
Aflibercept	9
Dexamethasone implant (Ozurdex^TM^)	2
Retinal laser coagulation	10

**Table 2:** Changes in key functional and anatomical parameters during the study. Overview of best-corrected visual acuity (BCVA), intraocular pressure (IOP), central subfield thickness (CST), retinal volumes (ETDRS 1 mm and 6 mm grids), and the proportion of eyes with intraretinal fluid (IRF) at baseline (mo0) and subsequent follow-ups (mo1, mo2, and mo3). Interquartile range (IQR) or standard error of the mean (SEM) is provided for each parameter, along with *p*-values comparing baseline to the end of the loading phase (mo3). Significant *p*-values in bold.

	BCVA (logMAR)	IOP (mmHg)	CST (µm)	Volume: ETDRS 1 mm (mm^3^)	Volume: ETDRS 6 mm (mm^3^)	IRF (Proportion of Eyes %)
mo0	0.20	16	325	0.26	9.05	100%
mo1	0.10	16	284	0.22	8.63	74%
mo2	0.10	16	278	0.22	8.47	58%
mo3	0.00	16	280	0.22	8.38	32%
IQR (mo0)	0.60	3	249	0.20	2.19	SEM: 0%
IQR (mo1)	0.40	7	77	0.06	1.07	SEM: 10%
IQR (mo2)	0.30	6	79	0.06	1.13	SEM: 12%
IQR (mo3)	0.30	6	40	0.03	0.99	SEM: 11%
*p* (mo0–mo3)	**<0.01**	**0.91**	**<0.01**	**<0.01**	**<0.01**	**<0.01**

**Table 3:** Correlation and regression analysis of best-corrected visual acuity (BCVA) with key anatomical biomarkers. Summary of the correlation coefficients (r), *p*-values, regression slopes, and y-intercepts for the relationship between BCVA and central subfield thickness (CST), as well as retinal volumes (Vol) within the 1 mm and 6 mm ETDRS grid circles. Significant *p*-values in bold.

BCVA (logMAR)	CST	Vol (ETDRS 1 mm Ring)	Vol (ETDRS 6 mm Ring)
r	0.58	0.57	0.49
*p*-value (correlation)	**<0.01**	**<0.01**	**0.02**
slope	0.0019 logMAR/µm	2.4180 logMAR/mm^3^	0.1616 logMAR/mm^3^
standard error (slope)	0.0004 logMAR/µm	0.5345 logMAR/mm^3^	0.0402 logMAR/mm^3^
Y-intercept	−0.43 logMAR	−0.43 logMAR	−1.22 logMAR
standard error (Y-intercept)	0.19 logMAR	0.19 logMAR	0.40 logMAR
R^2^	0.54	0.55	0.49
*p*-value (regression)	**<0.01**	**<0.01**	**<0.01**

## Data Availability

The datasets used and/or analyzed during the current study are available upon reasonable request.

## Abbreviations

ME	Macular Edema
RVO	Retinal Vein Occlusion
anti-VEGF	Anti-vascular endothelial growth factor
Ang-2	Angiopoietin-2
nAMD	Neovascular age-related macular degeneration
BCVA	Best-corrected visual acuity
CST	Central subfield thickness
OCT	Optical coherence tomography
IRF	Intraretinal fluid
SEM	Standard error of the mean
IQR	Interquartile range
IOP	Intraocular Pressure

## References

[1] Wong T.Y., Scott I.U. (2010). Clinical practice. Retinal-vein occlusion. N. Engl. J. Med..

[2] Laouri M., Chen E., Looman M., Gallagher M. (2011). The burden of disease of retinal vein occlusion: Review of the literature. Eye.

[3] Campochiaro P.A., Hafiz G., Shah S.M., Nguyen Q.D., Ying H., Do D.V., Quinlan E., Zimmer-Galler I., Haller J.A., Solomon S.D. (2008). Ranibizumab for macular edema due to retinal vein occlusions: Implication of VEGF as a critical stimulator. Mol. Ther..

[4] Schmidt-Erfurth U., Garcia-Arumi J., Gerendas B.S., Midena E., Sivaprasad S., Tadayoni R., Wolf S., Loewenstein A. (2019). Guidelines for the Management of Retinal Vein Occlusion by the European Society of Retina Specialists (EURETINA). Ophthalmologica.

[5] Haller J.A., Bandello F., Belfort R., Blumenkranz M.S., Gillies M., Heier J., Loewenstein A., Yoon Y.H., Jiao J., Li X.Y. (2011). Dexamethasone intravitreal implant in patients with macular edema related to branch or central retinal vein occlusion twelve-month study results. Ophthalmology.

[6] Goncalves M.B., Alves B.Q., Moura R., Magalhaes O., Maia A., Belfort R., de Avila M.P., Zas M., Saravia M., Lousas M. (2020). Intravitreal dexamethasone implant migration into the anterior chamber: A Multicenter Study From the Pan-American Collaborative Retina Study Group. Retina.

[7] Bhisitkul R.B., Blotner S., Steffen V., Haskova Z. (2020). Clinical trial versus real-world outcomes with anti-VEGF therapy for central retinal vein occlusion. Investig. Ophthalmol. Vis. Sci..

[8] Jumper J.M., Dugel P.U., Chen S., Blinder K.J., Walt J.G. (2018). Anti-VEGF treatment of macular edema associated with retinal vein occlusion: Patterns of use and effectiveness in clinical practice (ECHO study report 2). Clin. Ophthalmol..

[9] Saharinen P., Eklund L., Alitalo K. (2017). Therapeutic targeting of the angiopoietin-TIE pathway. Nat. Rev. Drug Discov..

[10] Regula J.T., Lundh von Leithner P., Foxton R., Barathi V.A., Cheung C.M., Bo Tun S.B., Wey Y.S., Iwata D., Dostalek M., Moelleken J. (2016). Targeting key angiogenic pathways with a bispecific CrossMAb optimized for neovascular eye diseases. EMBO Mol. Med..

[11] Joussen A.M., Ricci F., Paris L.P., Korn C., Quezada-Ruiz C., Zarbin M. (2021). Angiopoietin/Tie2 signalling and its role in retinal and choroidal vascular diseases: A review of preclinical data. Eye.

[12] Hattenbach L.O., Abreu F., Arrisi P., Basu K., Danzig C.J., Guymer R., Haskova Z., Heier J.S., Kotecha A., Liu Y. (2023). BALATON and COMINO: Phase III Randomized Clinical Trials of Faricimab for Retinal Vein Occlusion: Study Design and Rationale. Ophthalmol. Sci..

[13] Wykoff C.C., Abreu F., Adamis A.P., Basu K., Eichenbaum D.A., Haskova Z., Lin H., Loewenstein A., Mohan S., Pearce I.A. (2022). Efficacy, durability, and safety of intravitreal faricimab with extended dosing up to every 16 weeks in patients with diabetic macular oedema (YOSEMITE and RHINE): Two randomised, double-masked, phase 3 trials. Lancet.

[14] Heier J.S., Khanani A.M., Quezada Ruiz C., Basu K., Ferrone P.J., Brittain C., Figueroa M.S., Lin H., Holz F.G., Patel V. (2022). Efficacy, durability, and safety of intravitreal faricimab up to every 16 weeks for neovascular age-related macular degeneration (TENAYA and LUCERNE): Two randomised, double-masked, phase 3, non-inferiority trials. Lancet.

[15] Tadayoni R., Paris L.P., Danzig C.J., Abreu F., Khanani A.M., Brittain C., Lai T.Y.Y., Haskova Z., Sakamoto T., Kotecha A. (2024). Efficacy and Safety of Faricimab for Macular Edema due to Retinal Vein Occlusion: 24-Week Results from the BALATON and COMINO Trials. Ophthalmology.

[16] Vaz-Pereira S., Marques I.P., Matias J., Mira F., Ribeiro L., Flores R. (2017). Real-world outcomes of anti-VEGF treatment for retinal vein occlusion in Portugal. Eur. J. Ophthalmol..

[17] Kal M., Winiarczyk M., Gluszek S., Mackiewicz J. (2021). Choroidal thickness in lamellar macular holes. Graefes Arch. Clin. Exp. Ophthalmol..

[18] Kinyoun J., Barton F., Fisher M., Hubbard L., Aiello L., Ferris F. (1989). Detection of diabetic macular edema. Ophthalmoscopy versus photography--Early Treatment Diabetic Retinopathy Study Report Number 5. The ETDRS Research Group. Ophthalmology.

[19] Rehak M., Tilgner E., Franke A., Rauscher F.G., Brosteanu O., Wiedemann P. (2014). Early peripheral laser photocoagulation of nonperfused retina improves vision in patients with central retinal vein occlusion (Results of a proof of concept study). Graefes Arch. Clin. Exp. Ophthalmol..

[20] Michl M., Liu X., Kaider A., Sadeghipour A., Gerendas B.S., Schmidt-Erfurth U. (2021). The impact of structural optical coherence tomography changes on visual function in retinal vein occlusion. Acta Ophthalmol..

[21] Campochiaro P.A., Hafiz G., Mir T.A., Scott A.W., Solomon S., Zimmer-Galler I., Sodhi A., Duh E., Ying H., Wenick A. (2015). Scatter Photocoagulation Does Not Reduce Macular Edema or Treatment Burden in Patients with Retinal Vein Occlusion: The RELATE Trial. Ophthalmology.

[22] Iijima H. (2018). Mechanisms of vision loss in eyes with macular edema associated with retinal vein occlusion. Jpn. J. Ophthalmol..

[23] Callizo J., Ziemssen F., Bertelmann T., Feltgen N., Vogeler J., Koch M., Eter N., Liakopoulos S., Schmitz-Valckenberg S., Spital G. (2019). Real-World Data: Ranibizumab Treatment For Retinal Vein Occlusion In The OCEAN Study. Clin. Ophthalmol..

[24] Scott I.U., VanVeldhuisen P.C., Ip M.S., Blodi B.A., Oden N.L., Altaweel M., Berinstein D.M., Group S.I. (2018). Comparison of Monthly vs Treat-and-Extend Regimens for Individuals With Macular Edema Who Respond Well to Anti-Vascular Endothelial Growth Factor Medications: Secondary Outcomes From the SCORE2 Randomized Clinical Trial. JAMA Ophthalmol..

[25] Khodor A., Choi S., Nanda T., Caranfa J.T., Ruiz-Lozano R.E., Desai S.H., Liang M., Baumal C.R., Reed D.C., Cleary T.S. (2024). Visual and Anatomic Responses in Patients With Neovascular Age-Related Macular Degeneration and a Suboptimal Response to Anti-VEGF Therapy Switched to Faricimab. J. Vitreoretin. Dis..

